# Feasibility of software-based assessment for automated evaluation of tooth preparation for dental crown by using a computational geometric algorithm

**DOI:** 10.1038/s41598-023-39089-3

**Published:** 2023-07-22

**Authors:** Sangjun Han, Yuseung Yi, Marta Revilla-León, Burak Yilmaz, Hyung-In Yoon

**Affiliations:** 1 School of Dentistry, Seoul National University, Seoul, Republic of Korea; 2 Department of Prosthodontics, Seoul National University Dental Hospital, Seoul, Republic of Korea; 3 Department of Restorative Dentistry, School of Dentistry, University of Washington, Seattle, WA USA; 4 Research and Digital Dentistry, Kois Center, Seattle, WA USA; 5 Department of Prosthodontics, Tufts University, Boston, MA USA; 6 Department of Reconstructive Dentistry and Gerodontology, School of Dental Medicine, University of Bern, Bern, Switzerland; 7 Department of Restorative, Preventive and Pediatric Dentistry, School of Dental Medicine, University of Bern, Bern, Switzerland; 8 Division of Restorative and Prosthetic Dentistry, The Ohio State University, Columbus, OH USA; 9 Department of Prosthodontics, School of Dentistry and Dental Research Institute, Seoul National University, 101, Daehak-ro, Jongro-gu, Seoul, 03080 Republic of Korea

**Keywords:** Software, Fixed prosthodontics, Dental clinical teaching, Computational science

## Abstract

The purpose of this study was to propose the concept of software-based automated evaluation (SAE) of tooth preparation quality using computational geometric algorithms, and evaluate the feasibility of SAE in the assessment of abutment tooth preparation for single-unit anatomic contour crowns by comparing it with a human-based digitally assisted evaluation (DAE) by trained human evaluators. Thirty-five mandibular first molars were prepared for anatomical contour crown restoration by graduate students. Each prepared tooth was digitized and evaluated in terms of occlusal reduction and total occlusal convergence using SAE and DAE. Intra-rater agreement for the scores graded by the SAE and DAE and inter-rater agreement between the SAE and DAE were analyzed with the significance level (α) of 0.05. The evaluation using the SAE protocol demonstrated perfect intra-rater agreement, whereas the evaluation using the DAE protocol showed moderate-to-good intra-rater agreement. The evaluation values of the SAE and DAE protocols showed almost perfect inter-rater agreement. The SAE developed for tooth preparation evaluation can be used for dental education and clinical skill feedback. SAE may minimize possible errors in the conventional rating and provide more reliable and precise assessments than the human-based DAE.

## Introduction

Abutment tooth preparation should be performed in accordance with the fundamental principles of contemporary fixed prosthodontics for successful restorations^1^. The tooth structure should be preserved whenever possible, but optimal reduction is mandatory for restoration with a clinically acceptable prognosis^2^. Fixed restorations require sufficient reduction to achieve an appropriate thickness and shape with structural stability^3^. Tooth preparation with an optimal degree of taper is also essential to ensure proper retention and resistance of the fixed dental prosthesis and the absence of undercuts^4^.

One of the most critical components of clinical dental education is understanding the principles of tooth preparation in prosthodontics^5^. It is imperative that the student receives consistent and accurate feedback from the faculty members to improve their clinical performance before proceeding with actual patient care^5,6^. However, several factors contributed to disagreements in the evaluation of the students’ work, including subjective grading scales and insufficiently calibrated raters, which consequently failed to provide consistent and reliable feedback^6–8^. To address the factors that contribute to the lack of consistency in evaluation and promote more reliable assessment by faculty, the faculty calibration and well-defined grading criteria have been implemented^9^. Despite these improvements, inter-rater and intra-rater assessments by visual inspection with human eyes may not be consistent, and faculty members still frequently mark unacceptable student work as acceptable, and evaluation of the same work on different occasions has led to inconsistencies in grading being observed^10–12^.

To overcome these shortcomings, a human-based digitally assisted evaluation (DAE) using three-dimensional (3D) inspection and metrology software has been considered as an alternative which addresses the weaknesses of conventional visual inspection^5,13–15^. This method involves a thorough assessment by trained specialists, who evaluate the scanned data of abutment tooth preparation assisted by digital measurement with visually calculated scales^13–15^. Several studies have found that faculty evaluations using digital assessment software such as E4D Compare (E4D Technologies, Richardson, TX, USA), CEREC PrepCheck (Dentsply Sirona, Bensheim, Germany), and Prepassistant (Kavo, Biberach, Germany) show higher consistency than traditional assessment methods^5,6,8–13,16–19^. However, the inherent limitation of human-based evaluation remains a lack of consistency in the metrics manually assigned by raters, as well as discrepancies between raters^16–19^. Recently, a group of dental researchers and software engineers developed a novel software-based approach for the assessment of abutment tooth preparation with automated evaluation based on a computational geometric algorithm. Using software-based automated evaluation (SAE), the computational geometric algorithm determines the area to be evaluated and proceeds with automated evaluation, including digital measurements of the prepared tooth dimensions in a mathematically optimized model.

Therefore, the purpose of this study was to propose the concept of SAE of tooth preparation quality using computational geometric algorithms and to evaluate the feasibility of SAE in the assessment of abutment tooth preparation for single-unit anatomic contour crowns by comparing it with DAE by trained human evaluators. The null hypotheses of this study were: 1) there would be no differences in intra-rater agreement between the SAE and DAE, and 2) there would be no differences in the scores graded by the SAE and DAE.

## Results

The SAE operated robustly for all the prepared teeth, and the results in terms of the evaluation criteria are listed in Table 1. The average amount of occlusal reduction at the cusp tip was approximately 1.39 mm for the mesiobuccal (MB) cusp, 1.60 mm for the distobuccal (DB) cusp, 1.88 mm for the mesiolingual (ML) cusp, and 1.59 mm for the distolingual (DL) cusp. The average values of TOC were 26.44° and 18.60° in the mesiodistal (MD) and buccolingual (BL) planes, respectively. The average width of the preparation margin area was reported as 0.36 mm. The SAE assessment showed a complete agreement with the results.

**Table 1:** Assessment of tooth preparation quality by software-based automated evaluation (SAE).

Dataset no	Occlusal reduction (mm)				Axial wall taper $(^\circ )$			Margin width (mm)
	MB cusp	DB cusp	ML cusp	DL cusp		MD plane	BL plane	
1	1.09	1.28	1.65	1.40		25.03	9.81	0.38
2	1.65	2.03	2.10	1.74		39.21	40.39	0.33
3	1.32	1.75	1.59	1.52		27.89	10.52	0.42
4	1.93	1.92	2.70	1.92		25.92	21.76	0.26
5	1.59	1.76	1.66	1.72		29.51	21.89	0.31
6	1.19	1.67	1.77	1.84		35.52	27.02	0.29
7	2.12	2.62	2.04	1.61		39.11	31.25	0.38
8	1.31	1.57	2.18	1.68		26.00	14.09	0.34
9	1.42	1.65	1.67	1.51		31.47	13.32	0.37
10	1.00	1.64	1.63	1.16		34.71	27.10	0.45
11	1.25	1.42	1.41	1.24		33.70	14.05	0.32
12	1.18	0.98	1.67	1.52		25.31	13.97	0.46
13	1.14	1.30	2.16	1.60		19.02	24.57	0.26
14	1.02	1.07	1.61	1.01		23.29	09.36	0.35
15	1.59	2.18	1.32	1.04		22.42	15.54	0.42
16	1.65	1.70	2.55	2.22		21.25	17.06	0.55
17	1.49	2.10	2.49	1.99		28.96	19.02	0.28
18	1.35	1.31	2.06	1.39		21.84	14.08	0.18
19	1.35	1.22	2.19	1.93		19.70	17.09	0.51
20	1.67	1.70	2.26	1.87		31.29	12.68	0.24
21	1.03	1.21	1.53	1.25		19.74	22.71	0.40
22	1.06	1.16	1.51	1.72		23.96	25.79	0.31
23	1.23	1.53	1.70	1.61		22.25	00.79	0.36
24	0.61	0.86	1.92	1.42		18.12	03.55	0.42
25	1.51	1.83	2.14	1.62		23.90	19.31	0.37
26	1.04	1.38	1.46	1.40		30.26	28.30	0.36
27	1.61	1.95	1.91	1.82		33.83	30.04	0.40
28	0.97	0.64	1.16	0.73		23.43	17.80	0.23
29	1.41	1.37	1.65	1.58		12.84	20.41	0.32
30	1.19	1.45	1.45	1.41		23.45	11.98	0.30
31	1.82	2.09	2.09	1.66		25.07	25.20	0.30
32	2.21	2.26	2.24	1.79		31.45	30.89	0.39
33	1.74	1.85	2.65	2.35		18.69	38.33	0.63
34	2.07	2.19	2.26	2.13		22.27	11.24	0.28
35	0.81	1.09	1.36	1.15		34.68	14.25	0.41

*MB* mesiobuccal, *DB* distobuccal, *ML* mesiolingual, *DL* distolingual, *MD* mesiodistal, *BL* buccolingual.

The SAE and DAE scores for each criterion are listed in Table 2. The assessment using SAE showed identical scores in each round (1–3) of assessment. No significant differences were found between the rounds evaluated using the DAE for any of the evaluation criteria. Furthermore, there were no significant differences between SAE and DAE scores (p > 0.05).

**Table 2:** Scores of digitally assisted evaluation (DAE) and software-based automated evaluation (SAE) according to each criterion (mean ± standard deviation).

Protocol	DAE					SAE	
Evaluation round	#1	#2	#3	Mean	p-value^¶^	#1 (#2, #3)	p-value^†^
Occlusal reduction							
MB cusp	1.03 ± 0.45	1.17 ± 0.62	1.11 ± 0.58	1.11 ± 0.55	0.508	1.20 ± 0.58	0.373
DB cusp	1.20 ± 0.68	1.23 ± 0.60	1.14 ± 0.65	1.19 ± 0.64	0.861	1.26 ± 0.66	0.575
ML cusp	0.77 ± 0.73	0.97 ± 0.79	0.80 ± 0.76	0.85 ± 0.76	0.506	0.74 ± 0.74	0.479
DL cusp	1.20 ± 0.72	1.17 ± 0.62	1.14 ± 0.69	1.17 ± 0.67	0.924	1.23 ± 0.60	0.715
Axial taper							
MD plane	1.20 ± 0.83	1.14 ± 0.81	1.06 ± 0.73	1.13 ± 0.79	0.689	0.86 ± 0.69	0.061
BL plane	1.17 ± 0.82	1.26 ± 0.89	1.20 ± 1.83	1.21 ± 0.84	0.860	1.29 ± 0.83	0.646

*MB* mesiobuccal, *DB* distobuccal, *ML* mesiolingual, *DL* distolingual, *MD* mesiodistal, *BL* buccolingual.

^¶^p-value calculated from the Kruskal–Wallis H test for DAE evaluation values.

^†^p-value calculated from the Mann–Whitney test between SAE and DAE; statistically significant difference (p < .05) is marked with an asterisk (*).

**Figure 1:**
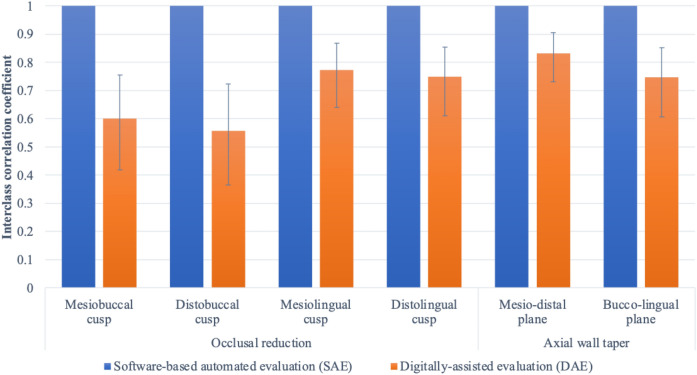
Intra-rater agreement (interclass correlation coefficient, Cronbach’s α) between scores graded using software-based automated evaluation (SAE) and scores graded by digitally assisted evaluation (DAE). Excellent: > 0.9; Good: 0.75–0.90; Moderate: 0.50–0.75; Poor reliability: < 0.5.

The intra-rater agreement for each evaluation method (SAE and DAE) is shown in Fig. 1. The SAE showed perfect agreement for every evaluation criterion. The DAE showed moderate-to-good intra-rater reliability, moderate reliability for the MB and DB cusps, BL TOC of axial wall taper assessments, good reliability for ML and DL cusps of occlusal reduction assessments, and MD TOC of axial wall taper assessments. None of these criteria exhibited excellent reliability.

**Figure 2:**
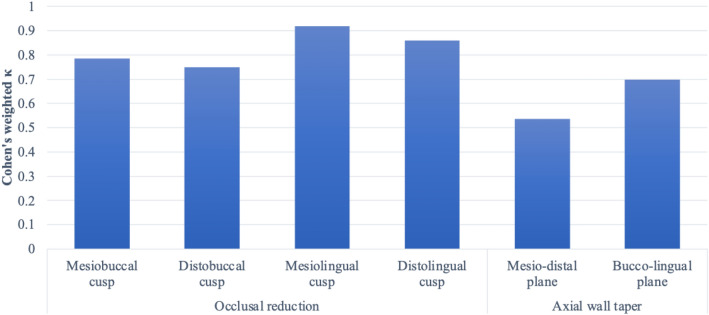
Inter-rater agreement [weighted Cohen’s kappa coefficient (κ)] between scores graded using software-based automated evaluation (SAE) and digitally assisted evaluation (DAE). Almost perfect: 0.81–1.00; Substantial: 0.61–0.80; Moderate: 0.41–0.60; Fair: 0.21–0.40; Slight: 0.01–0.20; and No agreement: ≤ 0.

The inter-rater agreements between the SAE and DAE scores differed depending on the evaluation criteria (Fig. 2). For occlusal reduction assessments, almost perfect agreement was found for the evaluation of the ML and DL cusps, and substantial agreement was found for the evaluation of the MB and DB cusps. For axial wall taper assessments, the evaluation of the BL TOC showed substantial agreement, whereas moderate agreement was found for the MD TOC assessments.

## Discussion

The results of this study support the validity of SAE in education and suggest the possibility of its clinical application in the assessment of tooth preparation for anatomic contour crowns. Intra-rater agreement showed that the faculty assessments using DAE were not always consistent, whereas the assessments with SAE showed perfect agreement for all evaluation criteria. Therefore, the first null hypothesis is rejected.

This study revealed that the evaluation consistency could be improved using SAE, which minimizes human-based biases or errors in measurement. In the DAE assessment, the evaluation of functional cusp (MB and DB cusps) reduction showed a lower intra-rater agreement than the other evaluation criteria. The mandibular buccal cusp preparation has a more complex shape than the other areas owing to the application of the functional cusp bevel. Thus, it is difficult for the faculty to consistently find the measurement reference point in the scanned preparation image through visual inspection. This weakness can be overcome with the use of mathematical algorithm-based software that finds a tangential line from the cusp tip on the anatomical tooth to the prepared tooth and defines the closest perpendicular location to the prepared tooth.

In addition to occlusal reduction in the cusp area and other evaluation criteria, consistent scores can be derived through the designation of measurement points based on the algorithm. The calculation of the weighted Cohen’s kappa between the SAE and DAE graded scores graded showed a moderate to almost perfect inter-rater agreement of > 0.5. Therefore, the second null hypothesis cannot be rejected; however, further evaluation is required in terms of other evaluation parameters, such as minimum reduction. Generally, the evaluator designates measuring points in the central fossa where the amount of reduction is anticipated to be the least. However, the actual minimum reduction is not always observed in the central fossa. Nevertheless, it was difficult to specify the point at which the minimum reduction could be detected by visual inspection. In future studies on SAE, the minimum reduction can be defined as the smallest value among the vertical distances from the anatomically intact tooth to the prepared tooth. A software-based assessment using a geometric algorithm may be used to find the point where the minimum reduction was made and can measure objective and precise values.

The traditional evaluation of tooth preparation relies on rater judgment based on visual inspection. Although current virtual assessment tools can improve objectivity and consistency in faculty evaluations, there remains significant subjectivity and a lack of inter-rater reliability^5,13^. This novel software-automated approach based on mathematical algorithms could eliminate the subjective intervention of raters and provide more reliable and precise assessments. SAE showed a high degree of agreement compared with human-based evaluation (DAE), demonstrating that SAE assessments can be used for both clinical evaluation and dental education instead of DAE.

Algorithm-based evaluations can improve the quality of restorative treatments by providing immediate quantitative feedback during abutment tooth preparation. Yamaguchi et al. introduced an algorithm-based evaluation method for predicting the debonding probability of resin composite crowns^20^. They developed a deep learning-based model utilizing a convolutional neural network (CNN) by training 6480 abutment images restored with resin composite crowns collected retrospectively. However, this prediction model may not be able to identify all abutment-related factors causing the debonding of crowns^20^. Using the geometric algorithm for this software-based evaluation, the numerical data of the prepared tooth related to the retention and resistance of the dental crown—such as the convergence angle and height of the abutment—can be calculated, and the possibility of crown debonding can be numerically evaluated by applying the verified criteria for each contributing factor^21–23^. Additionally, deep-learning-based dental crown design methods have recently been developed, using an algorithm that selects an appropriate design for many databases^24,25^. By integrating the computational geometric algorithm used in this study, the prepared abutment for crown restoration can be evaluated to predict the probability of failure, and used to design an anatomical contour crown with a better prognosis.

The SAE protocol with mathematical algorithms still has several limitations that need to be improved. Marginal integrity, damage to adjacent teeth, and quality of reduction (smoothness or waviness) cannot be measured at the current technological level. Another issue is the complexity of grading; the SAE assessment is based on evaluation criteria, and each score must be weighted to derive a final score. These limitations can be overcome through the development of algorithms involved, and further research should be conducted to apply various evaluation criteria. Within the limitations of this feasibility study, the SAE developed for the assessment of abutment tooth preparation can be used in clinical dental education. This SAE can minimize possible errors in conventional human-based evaluations and provide more reliable and precise assessments than DAE.

## Materials and methods

### Data acquisition of abutment tooth geometry

A standardized mandibular right first molar acrylic resin tooth (Simple Root Tooth Model A5A-200; Nissin Dental Products, Kyoto, Japan) was used to develop the SAE. The resin tooth was prepared in accordance with the requirements for anatomic contour crowns with a 1.5-mm occlusal reduction, 1–1.5-mm axial reduction with rounded internal line angles, and 1-mm circumferential chamfer finish line. The prepared abutment was then digitized under ambient light condition by an intraoral scanner (i500, iScan version 1.2.0.1; Medit, Seoul, South Korea) with a reported precision of 25-µm, by a single evaluator with 10 years of clinical experience in digital dentistry and the scan was stored in standard tessellation language file (STL) format. The scanner was calibrated according to the manufacturer’s instructions, and calibration was performed for each scan process. As the intraoral scanner has been reported to have a similar level of congruence in 3D data as a laboratory scanner, digitization with the intraoral scanner may also be considered reliable^26,27^. The scanning process for each abutment was performed continuously in a single attempt, based on a reliability map with display mode, to verify complete scanning^28^. Before tooth preparation, the original anatomical form of the resin molar was recorded as a reference for the tooth preparation assessment. Subsequently, the 3D scan data of the abutment resin tooth (before and after tooth preparation) were used to develop the SAE using a computational geometric algorithm and mathematical modelling.

### Detection of tooth preparation margin

**Figure 3:**
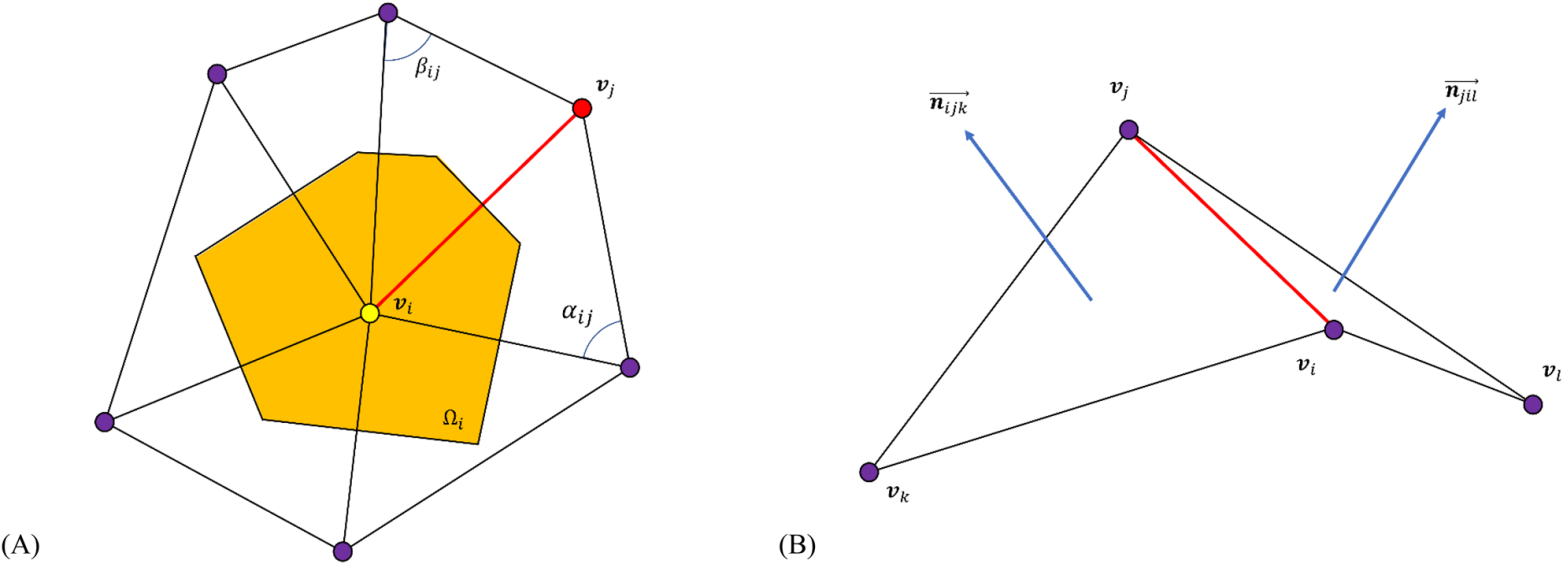
Schematic diagram of algorithms to define marginal location of tooth preparation. (**A**) Laplacian with cotangent weights, which approximates mean curvature of vertices on a piecewise flat surface, was used to find boundary vertices on the preparation surface. (**B**) When connecting vertices at marginal locations, edge length was adjusted to represent sharpness of edge. By taking advantage of adjusted edge length, the calculated path accurately followed features of located preparation margins.

First, the axial wall evaluation area was defined by identifying the preparation margins. The three-dimensional scan data were obtained using the piecewise flat surface method, using geometrical properties that represent the ‘sharpness’ can find the preparation margin. Sharpness can be observed in two aspects: sharpness at each vertex and sharpness at each edge. The sharpness of each vertex is expressed as a Laplacian that is related to the surrounding vertices (Eq. 1, Fig. 3A), and sharpness at the edge is expressed as the relationship between the two faces near the edge (Eq. 2, Fig. 3B)^29^.

1 $${\left(\mathrm{\Delta f}\right)}_{\mathrm{i}}=\frac{1}{{\Omega }_{\mathrm{i}}}{\sum }_{\left(\mathrm{i},\mathrm{j}\right)\in\upvarepsilon }\frac{\mathrm{cot}{\mathrm{\alpha }}_{\mathrm{ij}}+\mathrm{cot}{\upbeta }_{\mathrm{ij}}}{2}\left({\mathrm{v}}_{\mathrm{i}}-{\mathrm{v}}_{\mathrm{j}}\right)$$

2 $${\mathrm{e}}_{\mathrm{ij}}^{\mathrm{^{\prime}}}=\frac{\left(\overrightarrow{{\mathrm{n}}_{\mathrm{ijk}}}\cdot \overrightarrow{{\mathrm{n}}_{\mathrm{jil}}}+1\right)}{2\left|\overrightarrow{{\mathrm{n}}_{\mathrm{ijk}}}\right|\left|\overrightarrow{{\mathrm{n}}_{\mathrm{jil}}}\right|}{\mathrm{e}}_{\mathrm{ij}}$$

**Figure 4:**
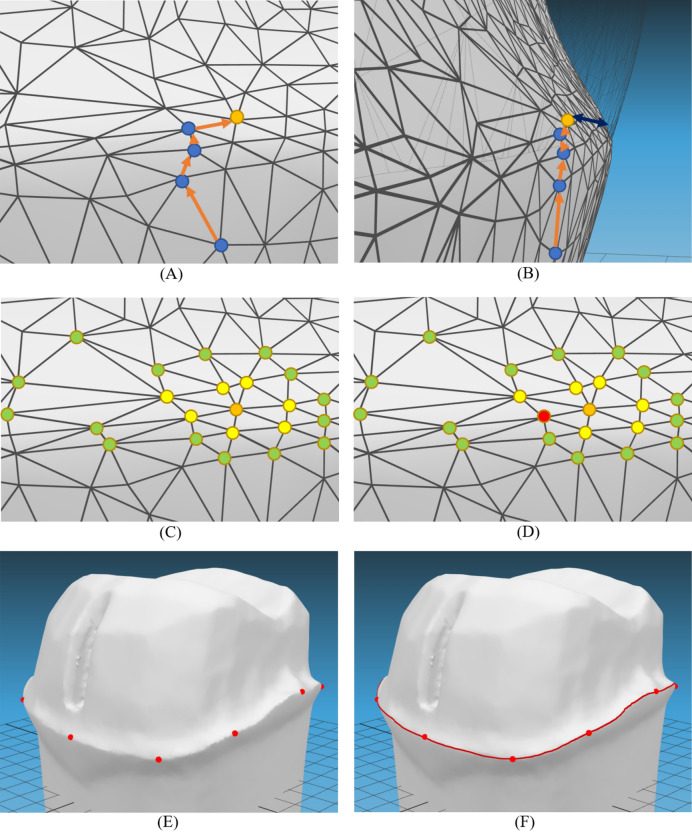
Process of defining tooth preparation margin based on geometric algorithm. When the shortest distance to the tooth model was smaller than the error bound, each search point was updated with the vertex located on the crown area (blue vertices). When the shortest distance was bigger than the threshold (orange vertex and dark blue arrow), it was assumed to be around the preparation margin (**A**,**B**). Among the neighborhood vertices (yellow and green), the vertex with the biggest Laplacian value (red) was determined as the vertex on the preparation margin (**C**,**D**). By connecting the intentionally distorted distance field, the preparation margin can be mathematically detected (**E**,**F**).

**Figure 5:**
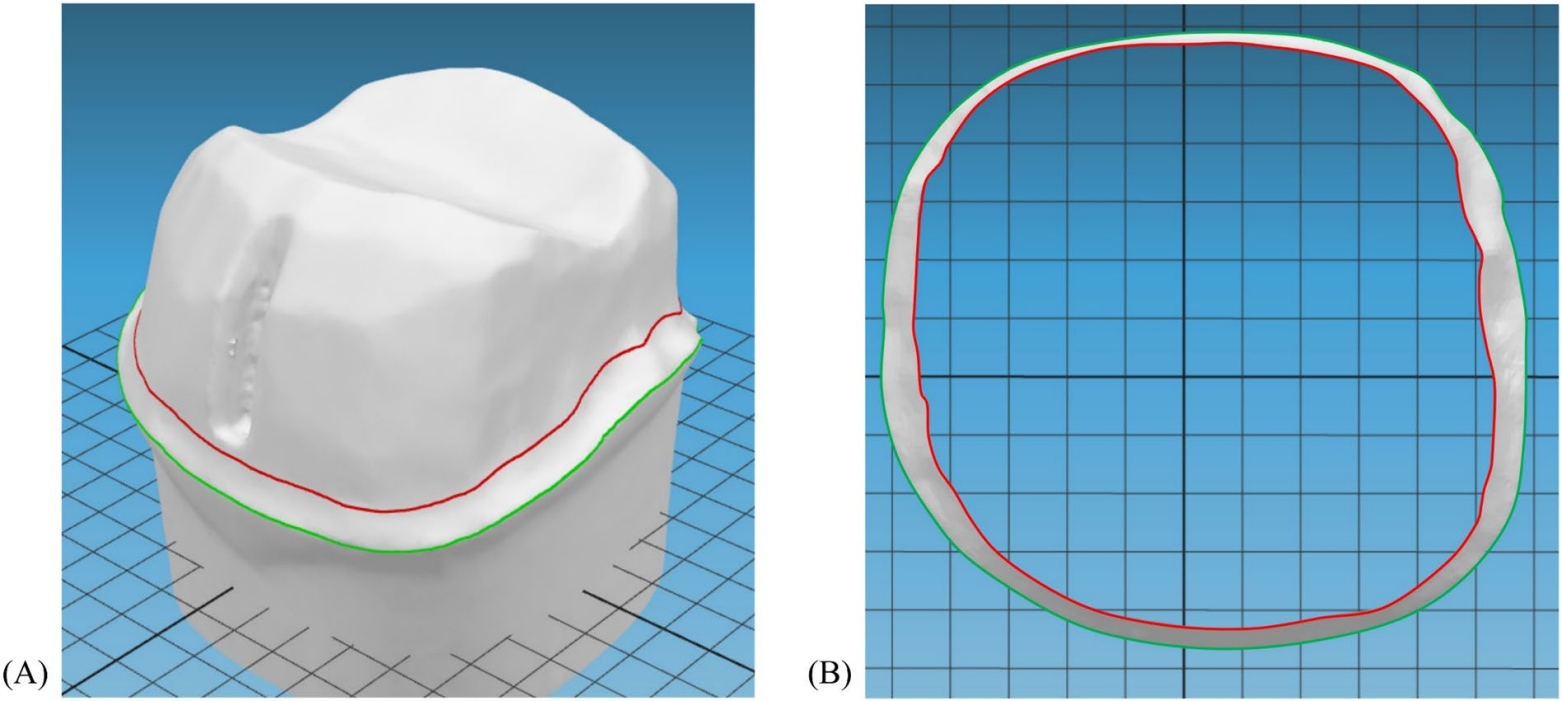
Process of defining width of preparation margin area. (**A**) Width of preparation margin area can be defined as a circular band-shape region above the preparation margin (green line) until axial wall area begin (red line). (**B**) Average width of margin area can be approximately calculated from the outer perimeter (green line), inner perimeter (red line), and area between the perimeters generated by projecting the margin in the direction of the path of insertion.

The images of both the anatomically intact and prepared tooth models were registered, and the software determined 12 initial search vertices in the root area. The shortest distance between the initial search vertices of the anatomical tooth model and the prepared tooth was calculated. If the shortest distance was smaller than the error bound (ε), the search point was updated with the vertex located in the crown area (Fig. 4A,B), and the calculation of the shortest distance from the new search point to the prepared tooth was repeated. If a position was found beyond ε, it was considered to be at the preparation margin (Fig. 4C). By comparing the Laplacian values of adjacent vertices, the vertex with the largest Laplacian value was determined as the vertex on the tooth preparation margin (Fig. 4D). To determine the preparation margin more accurately, the dimensions were intentionally distorted by closely adjusting the distances around the sharp edges. The final tooth preparation margin was determined mathematically by identifying the shortest path connecting all 12 vertices (Fig. 4E,F). The preparation margin area was defined as the circular band-shaped area above the preparation margin until the axial wall area began. The approximate average preparation margin width was calculated by projecting the margin area in the path of insertion of the prosthesis to form a flat band-shaped area and dividing the band area by the average length of the long and short perimeters (Fig. 5).

### Definition of the axial reduction area

**Figure 6:**
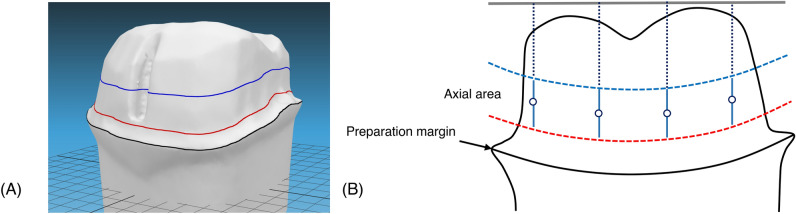
Process of defining the axial area of prepared tooth based on the preparation margin area. (**A**) The lower (red line) and upper (blue line) boundaries of axial area of prepared tooth were geometrically generated from the location of the preparation margin (black line). (**B**) The lower boundary (dashed red line) was defined by finding the intersection polylines between the prepared tooth model and translating surface generated by the preparation margin. The upper boundary (dashed blue line) was defined as the intersection between the prepared tooth model and the curved surface generated by group of vertices that internally divide the points of the vertices on the surface generating the lower boundary and flat surface on the occlusal side (solid grey line).

The upper boundary of the preparation margin area was defined by finding the intersection polylines between the prepared tooth and translating surface generated by the preparation margin (Fig. 6A). The lower boundary of the axial area is defined as the area directly above the preparation margin. When defining the upper boundary of the axial wall area, the height of the axial wall can differ for each prepared surface (buccal, lingual, mesial, and distal). Generally, the axial wall height of the buccal and lingual surfaces is greater than that of the mesial and distal surfaces. Considering this anatomic condition, the upper boundary of the axial wall was defined as the intersection between the prepared tooth model and the curved surface generated by a group of vertices that internally divided the points of the vertices on the surface, generating the lower boundary (Fig. 6B, dotted red line) and flat surface on the occlusal side (Fig. 6B, black line).

**Figure 7:**
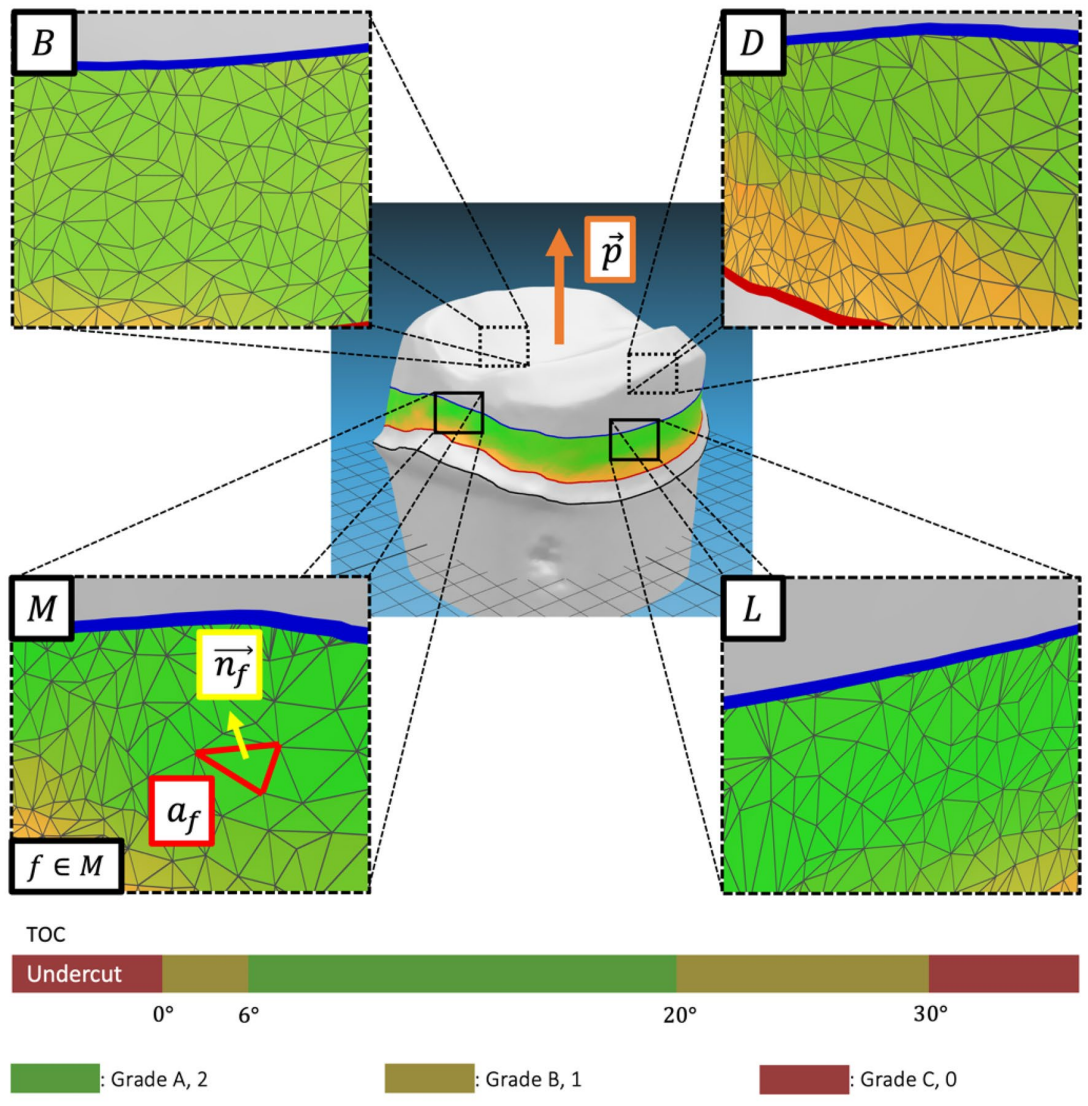
Representative image of total occlusal convergence (TOC) calculation algorithm. $\overrightarrow{p}$ is direction of insertion path, ${a}_{f}$ is area of face *f*, and $\overrightarrow{{n}_{f}}$ is normal direction of ${a}_{f}$. Taper T is calculated for each surface (Mesial, Distal, Buccal, Lingual) using area weighted average taper. TOC for the mesiodistal (MD) plane is calculated by adding Taper of M and Taper of D, and TOC for buccolingual (BL) plane is calculated by adding Taper of B and Taper of L. Grades A (2 points, green, ‘Acceptable’), B (1 point, yellow, ‘Marginally acceptable’), and C (0 point, red, ‘Unacceptable’) were color-marked, based on evaluation criteria for tooth preparation.

The axial wall area was divided into buccal $(\mathrm{B})$, lingual $(\mathrm{L})$, mesial $(\mathrm{M})$, and distal $(\mathrm{D})$ areas. Total occlusal convergence (TOC) was obtained by summing the average tapers of the contralateral area: buccal (B) and lingual (L) average tapers, and mesial (M) and distal (D) average tapers (Fig. 7). The average taper was calculated using the following equation (Eq. 3):

3 $$\begin{gathered} {\text{T}}_{{{\text{B}},{\text{L}},{\text{M}},{\text{D}}}} = 90^\circ - \frac{1}{{{\text{A}}_{{{\text{B}},{\text{L}},{\text{M}},{\text{D}}}} }}\mathop \sum \limits_{{{\text{f}} \in {\text{B}},{\text{ L}},{\text{ M}},{\text{ D}}}} {\text{a}}_{{\text{f}}} \cos^{ - 1} \frac{{{\vec{\text{p}}} \cdot \overrightarrow {{{\text{n}}_{{\text{f}}} }} }}{{\left| {{\vec{\text{p}}}} \right|\left| {\overrightarrow {{{\text{n}}_{{\text{f}}} }} } \right|}}, {\text{A}}_{{{\text{B}},{\text{L}},{\text{M}},{\text{D}}}} = \mathop \sum \limits_{{{\text{f}} \in {\text{B}},{\text{L}},{\text{M}},{\text{D}}}} {\text{a}}_{{\text{f}}} \hfill \\ {\text{TOC}}_{{{\text{BL}}}} = {\text{T}}_{{\text{B}}} + {\text{T}}_{{\text{L}}} , {\text{TOC}}_{{{\text{MD}}}} = {\text{T}}_{{\text{M}}} + {\text{T}}_{{\text{D}}} \hfill \\ \end{gathered}$$

The average taper $\mathrm{T}$ was defined as the sum of the area-weighted taper, and the taper of each face $\mathrm{f}$ was calculated using the path of insertion $\overrightarrow{\mathrm{p}}$, area of the face ${\mathrm{a}}_{\mathrm{f}}$, and the sum of the area of the face ${\mathrm{a}}_{\mathrm{f}}$ for each side (buccal, lingual, mesial, and distal) ${A}_{B,L,M,D}$, and normal of the face $\overrightarrow{{\mathrm{n}}_{\mathrm{f}}}$. The TOC of the buccolingual and mesiodistal planes was evaluated as the sum of the opposing average tapers.

### Definition of occlusal reduction area

**Figure 8:**
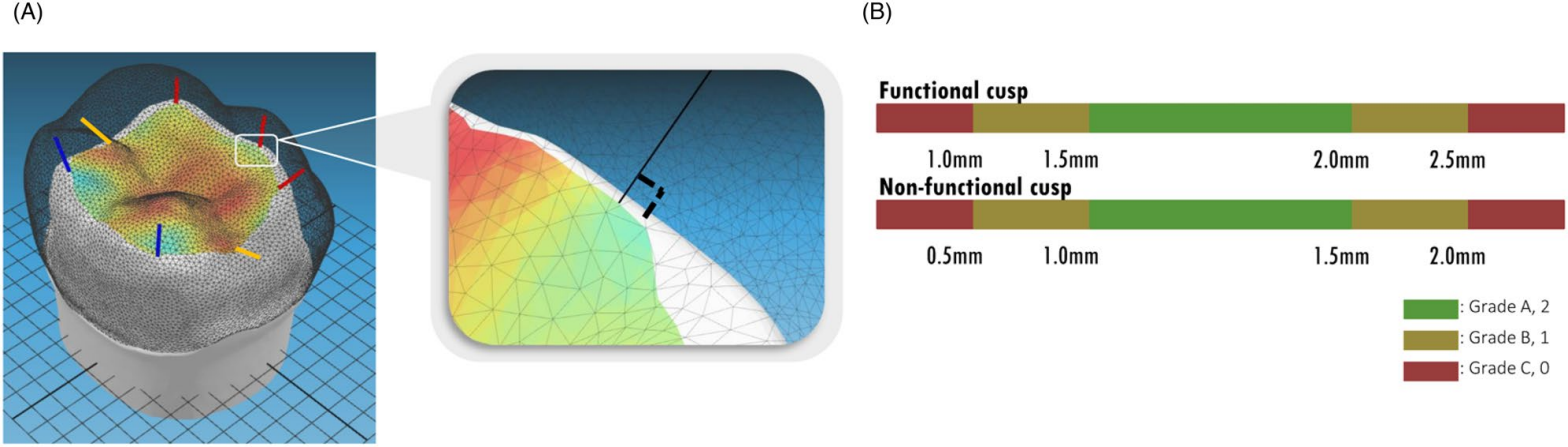
(**A**) Process of defining occlusal area and implementing an optimized algorithm to evaluate occlusal reduction dimension. Cusp reduction was defined as shortest distance from cusp tip of anatomical tooth model to that of prepared tooth. (**B**) Grades A (2 points, green, ‘Acceptable’), B (1 point, yellow, ‘Marginally acceptable’), and C (0 point, red, ‘Unacceptable’) were color-marked, based on the evaluation criteria for tooth preparation.

Seven standard points (each on the five cusp tips and two marginal ridge areas) were identified on the occlusal surface of each abutment tooth with an intact anatomical structure (before preparation). A geometric line was drawn from each standard point to the corresponding point on the occlusal surface of the prepared tooth, allowing the formation of a line perpendicular to the prepared surface. The boundary formed by the intersecting points on the occlusal surface of the prepared tooth passing through the closest path between the perpendicular lines was used to define the occlusal reduction area of the prepared tooth (Fig. 8A). Cusp reduction was defined as the shortest distance from the cusp tip of the anatomical tooth model to that of the prepared tooth model. To optimize the process of determining the shortest distance, a bounding volume hierarchy and priority queue were used^30^.

### Establishment of color-coded grade system for SAE

After the development of the SAE using a computational geometric algorithm and mathematical modelling, the grading system of the SAE was established according to the criteria for tooth preparation assessment (Table 3). The assessment scores were visualized and color-coded into three different grades: A (green), B (yellow), and C (red), according to the evaluation criteria (Fig. 8B). The points assigned to each grade category were: 2 points for grade A (acceptable), 1 point for grade B (marginally acceptable), and 0 point for grade C (unacceptable).

**Table 3:** Evaluation criteria of abutment tooth preparation for anatomic contour crown restoration.

Evaluation criteria (point)	Acceptable (2)	Marginally acceptable (1)	Unacceptable (0)
Occlusal reduction			
Mesiobuccal (MB) cusp	1.5–2.0 mm	1.0–1.5 mm or 2.0–2.5 mm	< 1.0 mm or > 2.5 mm
Distobuccal (DB) cusp	1.5–2.0 mm	1.0–1.5 mm or 2.0–2.5 mm	< 1.0 mm or > 2.5 mm
Mesiolingual (ML) cusp	1.0–1.5 mm	0.5–1.0 mm or 1.5–2.0 mm	< 0.5 mm or > 2.0 mm
Distolingual (DL) cusp	1.0–1.5 mm	0.5–1.0 mm or 1.5–2.0 mm	< 0.5 mm or > 2.0 mm
Axial wall taper (total occlusal convergence, TOC)			
Mesiodistal (MD) plane	TOC 6°–20°	TOC 0°–6° or 20°–30°	TOC > 30° or < 0° (undercut)
Buccolingual (BL) plane	TOC 6°–20°	TOC 0°–6° or 20°–30°	TOC > 30° or < 0° (undercut)

### Feasibility of SAE for tooth preparation assessment: comparison with DAE

A total of 35 mandibular right first molar resin teeth (Simple Root Tooth Model A5A-200; Nissin Dental Products, Kyoto, Japan), which had been prepared by thirty-five graduate dental students and submitted to as part of the fulfillment for routine practical examination, were retrospectively collected and completely anonymized. Evaluation and approval by an institutional review board was not required. Each resin tooth was carefully prepared according to the requirements of the anatomical contour crown restoration. Each prepared abutment tooth was then digitized under ambient light conditions using an intraoral scanner (i500, iScan version 1.2.0.1; Medit, Seoul, South Korea) by a single evaluator with 10 years of clinical experience in digital dentistry and stored in STL format. Considering the limited angle and focal distance of the small target area, an intraoral scanner was used to digitize the tooth^31,32^. Before each scanning procedure, the scanner was calibrated according to the manufacturer’s instructions.

For SAE assessment, the scan data of each prepared tooth were uploaded and automatically assessed in terms of tooth preparation quality using the software tested in this study. All the necessary information for the evaluation criteria for tooth preparation was displayed with no intervention by human evaluators. The reported data are presented according to the evaluation criteria (Table 3).

For the assessment using DAE, each prepared tooth was assessed by two board-certified prosthodontists who were full-time faculty members and had worked as evaluators of practical examinations for graduate students, by using inspection and metrology software (Medit Compare, Medit, Seoul, South Korea). This software was designed to analyze, align, measure (including distance, area, length, and angle), and compare 3D data. The human-based evaluation was conducted three times, with a washout period of one week interval between each evaluation. In each round, the prepared teeth were randomly assigned to an evaluator (Research Randomizer; https://www.randomizer.org/). Tooth preparation quality was scored identically to the SAE system according to each evaluation criterion (Table 3). The score for each quality assessment was determined based on the agreement between the two evaluators. For each assessment, the scanned data of the abutment tooth with intact occlusal anatomy (before preparation) and those of the prepared abutment tooth were superimposed using inspection software (Medit Compare). Superimposition of the two datasets was conducted after verifying the alignment in the synchronized coordinate space. Using ‘Align with selected area’ function, the initial alignment was performed by manually designating the same reference area for each scan data, and 3D superimposition was completed by the best-fit alignment using iterative closest point algorithm between the point clouds of digitized data. Each evaluator carefully marked the measurement points to evaluate the superimposed scan data. The distance or angle between the points was measured virtually based on the location of the marked points. For the occlusal reduction evaluation, a human evaluator designated each cusp tip and central fossa on the reference scan image of the abutment tooth with intact occlusal anatomy. By connecting the designated cusp tip and the corresponding cusp point on the scan image of prepared tooth, the inspection software (Medit Compare) tangentially builds a 2-dimensional plane passing through this line and measures the distance by using the ‘Measure distance by two points’ function. The distance between the cusps of the abutment tooth before preparation and those of the prepared tooth was used as the amount of occlusal reduction. The reduction was scored based on the evaluation criteria listed in Table 3. The axial wall taper was evaluated using the ‘Create sections’ and ‘Measure angle by four points’ functions in the software. A human evaluator marked the reference lines that passed vertically through the center of each buccal and lingual surface of the scan data of the prepared tooth along the long axis. The software automatically calculated the angle between these lines as the TOC values in the BL and MD planes, and the measured values were scored according to evaluation criteria (Table 3).

### Statistical analysis

The intra-rater agreement of the three evaluation scores for each criterion graded using SAE and DAE was analyzed by calculating the interclass correlation coefficient (ICC, Cronbach’s α). An ICC value of > 0.9 was considered to indicate excellent reliability, while 0.75–0.90 indicated good; 0.50–0.75 indicated moderate; and < 0.5 indicated poor reliability^33^. The inter-rater agreement between the scores from the SAE and DAE protocols was analyzed by calculating the weighted Cohen’s kappa coefficient (κ) for each evaluation criterion. A Cohen’s κ value of 0.81–1.00 was considered almost perfect agreement; 0.61–0.80 was considered substantial; 0.41–0.60 was considered moderate; 0.21–0.40 was considered fair; 0.01–0.20 was considered slight; and ≤ 0 was considered no agreement^34^. Statistical analyses were performed using the R software (ver. 4.1.2), with a significance level (α) of 0.05.

## Data Availability

The datasets generated and/or analyzed in the current study are available from the corresponding author upon reasonable request.
